# Knowledge, Attitudes, and Practices (KAP) of Postgraduate Medical Trainees Regarding Patient Care in Diabetes and Hypertension

**DOI:** 10.7759/cureus.76131

**Published:** 2024-12-21

**Authors:** Imran Qadar Khattak, Mustaqeem Shah, Muhammed Irfan, Salman Shah, Muhammad Umer, Mubashir Qadar Khattak, Qandeel Murtaza, Riffat Shaheen, Ayesha Usman

**Affiliations:** 1 Internal Medicine, Hayatabad Medical Complex, Peshawar, PAK; 2 Internal Medicine, Lady Reading Hospital, Peshawar, PAK; 3 Medicine and Surgery, Khyber Girls Medical College, Peshawar, PAK

**Keywords:** attitudes, diabetes management, hypertension management, knowledge, postgraduate medical trainees, practices

## Abstract

Background: Diabetes and hypertension are common chronic illnesses that need to be effectively managed. To improve patient outcomes, healthcare workers' knowledge, attitudes, and practices (KAP) are essential.

Objective: This study aimed to evaluate the KAP of postgraduate medical trainees regarding the management of diabetes and hypertension.

Methodology: A cross-sectional study was conducted over one year from January 2023 to December 2023. The study participants were 384 postgraduate medical trainees who fulfilled the predetermined inclusion and exclusion criteria. A validated questionnaire that evaluated attitudes toward patient-centered care, knowledge of clinical guidelines, and illness management methods was used to gather data. SPSS version 26 (IBM Corp., Armonk, NY) was used for statistical analysis to investigate correlations between KAP levels and demographic characteristics.

Results: Of the 384 trainees, 320 (83.33%) correctly identified clinical guidelines for diabetes management and 290 (75.75%) for hypertension management. The majority (350 (91.67%)) supported patient-centered care, with 320 (83.33%) believing that patients should be actively involved in treatment decisions. In terms of practices, 300 (78.13%) regularly monitored blood glucose levels, and 320 (83.33%) routinely checked blood pressure in hypertensive patients. Adherence to national treatment guidelines was reported by 280 (72.92%) for diabetes and 270 (70.31%) for hypertension. Younger trainees and those with 6-12 months of clinical experience exhibited higher KAP scores. Significant associations were found between age, clinical experience, and training program and KAP levels.

Conclusion: Although postgraduate trainees show strong knowledge of and favorable attitudes toward managing diabetes and hypertension, there is room for improvement in their practical application, particularly with regard to medication adherence and following guidelines, in order to provide high-quality patient care.

## Introduction

As two of the most common chronic diseases in the world, diabetes and hypertension present serious public health issues because of their consequences and strain on healthcare systems [1,2]. Since they together contribute significantly to avoidable morbidity and death, managing them effectively requires a multifaceted strategy that includes medical expertise, optimistic outlooks, and evidence-based therapeutic procedures [3]. In this regard, the provision of optimum treatment by healthcare professionals, especially postgraduate medical trainees, is essential to improve patient outcomes [4].

In hospital and clinic settings, postgraduate medical trainees are often the first point of contact for patients, acting as a vital bridge between primary healthcare and specialist care [5,6]. Their knowledge of diabetes and hypertension, attitudes toward patient-centered treatment, and compliance with suggested clinical recommendations are all critical to their capacity to successfully manage these illnesses [7]. However, even among highly qualified healthcare professionals, evidence indicates that knowledge gaps, attitudes, and inconsistent behaviors might make it difficult to offer high-quality treatment [8,9]. Patient outcomes are strongly impacted by trainees' proficiency in treating chronic illnesses such as diabetes and hypertension as they move from classroom instruction to real-world clinical applications [10,11].

A thorough grasp of how postgraduate trainees use their knowledge and attitudes in actual clinical situations is still lacking, despite improvements in medical education. The majority of the research now in publication concentrates on theoretical understanding or clinical results, with little attention paid to the interaction of knowledge, attitudes, and practices (KAP) in the treatment of long-term illnesses such as diabetes and high blood pressure. Additionally, research often ignores the particular difficulties that trainees encounter in environments with limited resources, such as high patient numbers and a lack of resources, which may have a big impact on their practices.

Closing the gap between medical education and practice requires addressing this research gap. A thorough assessment of trainees' KAP may help guide specific enhancements in postgraduate training and increase their capacity to provide high-quality treatment for long-term illnesses.

Research objective

The objective of this study was to evaluate the KAP of postgraduate medical trainees in managing diabetes and hypertension.

## Materials and methods

Study design and setting

This cross-sectional study was conducted at the Hayatabad Medical Complex (HMC), Peshawar, from January 2023 to December 2023.

Inclusion and exclusion criteria

Postgraduate medical trainees who were enrolled in any clinical training program, had at least six months of clinical experience, and were willing to provide informed permission were all eligible to participate in the research. Those who had already had specialized training in cardiology or endocrinology, trainees on prolonged leave throughout the research period, and those who were hesitant to participate or who provided incomplete questionnaire replies were excluded.

Sample size

A total of 384 postgraduate medical trainees were included in this study, selected using a simplified stratified random sampling approach to ensure representation across all clinical specialties. The study was conducted across three major healthcare institutions in Peshawar: Hayatabad Medical Complex, Lady Reading Hospital, and Khyber Teaching Hospital. To ensure feasibility and adequate subgroup representation, the sample size represented 10% of the total postgraduate trainees enrolled across all specialties in these hospitals. Participants were stratified by specialties, such as internal medicine, surgery, pediatrics, and gynecology, to maintain a balanced representation across disciplines. From each stratum, trainees were randomly selected using lists provided by the hospital administrations, minimizing selection bias and ensuring equal inclusion of eligible participants.

To capture the diverse characteristics of postgraduate medical trainees, the sample deliberately included participants with varying levels of clinical experience and expertise. This stratified approach accounted for the heterogeneity typical of postgraduate training programs, enabling a more comprehensive evaluation of their knowledge, attitudes, and practices (KAP) in managing diabetes and hypertension. By encompassing this diversity, the study aimed to provide robust insights into the preparedness of trainees across a broad spectrum of clinical training settings.

Data collection

A systematic, self-designed questionnaire was used to gather data on participants' knowledge of best practices for managing hypertension and diabetes. The questionnaire was validated through a pilot test and expert review to ensure content accuracy and relevance. It consisted of three components, which were disseminated both digitally and on paper: practices, which assessed practical clinical management techniques such as medication adherence and patient counseling; attitudes, which assessed views of patient-centered care, adherence to evidence-based practices, and obstacles to effective management; and knowledge, which included questions about clinical guidelines, diagnostic criteria, and treatment strategies. Before submission, trained research assistants ensured that the surveys were complete. The data collection method was anonymous, and participation was entirely voluntary. To further assess participants' practical clinical performance, we calculated efficiency rates based on correct responses in the knowledge section of the survey. The efficiency rate was determined as the proportion of correct answers to total questions in key domains, such as diagnostic criteria and medication guidelines for diabetes and hypertension. This provided a quantitative measure of trainees' ability to apply knowledge effectively, which adds depth to the analysis of their knowledge, attitudes, and practices.

Statistical analysis

The collected data were analyzed using SPSS version 26 (IBM Corp., Armonk, NY). Descriptive statistics, including frequencies, means, and standard deviations, were employed to summarize demographic information and responses related to KAP. Inferential statistical tests, such as the chi-square test of independence, were conducted to identify potential relationships between KAP levels and various demographics (e.g., age and gender) and professional factors (e.g., clinical experience and educational level). Statistical significance was defined as a p-value < 0.05, ensuring a robust interpretation of the findings. All analyses adhered to rigorous data-handling standards, and results were presented in detailed tabular formats for clarity and precision.

Ethical approval

The research received ethical clearance from the HMC Ethical Review Committee (approval number: 132/OD/HMC/2022). All subjects provided written informed permission before being included in the research, and confidentiality was maintained.

## Results

The research included 384 postgraduate medical trainees, with 20.83% under 25 years, 33.44% between 31 and 35 years, and 24.48% over 35 years (Table 1). The majority of the trainees (31.25%) were between 25 and 30 years old. There were 41.67% male trainees and 58.33% female trainees. The majority of the participants (52.08%) had clinical experience ranging from six to 12 months, followed by those with 13-24 months of clinical experience (31.25%) and more than 24 months of clinical experience (16.67%). General medicine training accounted for 39.06% of the total, followed by surgery (26.04%), pediatrics (21.88%), and other specialties (13.02%). Educational backgrounds were divided between postgraduates (Doctor of Medicine (MD)/Master of Surgery (MS), 42.71%) and graduates (Bachelor of Medicine, Bachelor of Surgery (MBBS), 57.29%). Furthermore, 31.25% had three to four years of training, 16.67% had more than five years, and 52.08% had been in their first two years.

**Table 1:** Demographic Characteristics of Postgraduate Medical Trainees MBBS: Bachelor of Medicine, Bachelor of Surgery, MD: Doctor of Medicine, MS: Master of Surgery

Variable		Number of patients (number (%))
Age group	<25 years	80 (20.83)
	25-30 years	120 (31.25)
	31-35 years	90 (23.44)
	>35 years	94 (24.48)
Gender	Male	160 (41.67)
	Female	224 (58.33)
Clinical experience	6-12 months	200 (52.08)
	13-24 months	120 (31.25)
	>24 months	64 (16.67)
Training program	General medicine	150 (39.06)
	Surgery	100 (26.04)
	Pediatrics	84 (21.88)
	Other	50 (13.02)
Educational level	Graduate (MBBS)	220 (57.29)
	Postgraduate (MD/MS)	164 (42.71)
Years of training	1-2 years	200 (52.08)
	3-4 years	120 (31.25)
	5+ years	64 (16.67)

According to the knowledge test, 75.75% of the trainees correctly recognized clinical recommendations for hypertension care and 83.33% for diabetes management (Table 2). With 80.83% for hypertension and 72.92% for diabetes, there was a high level of comprehension of the diagnostic criteria. First-line medicine knowledge was somewhat lower, with 70.31% correctly recognizing diabetic therapies and 67.71% properly identifying hypertension treatments. With 85.42% of the respondents correctly identifying lifestyle changes for controlling both illnesses, this task had the greatest accuracy.

**Table 2:** Knowledge of Postgraduate Trainees on Diabetes and Hypertension Management

Knowledge question	Correct responses (number (%))
Clinical guidelines for diabetes management	320 (83.33)
Clinical guidelines for hypertension management	290 (75.75)
Diagnostic criteria for diabetes	280 (72.92)
Diagnostic criteria for hypertension	310 (80.83)
First-line medications for diabetes	270 (70.31)
First-line medications for hypertension	260 (67.71)
Lifestyle modifications for managing both conditions	330 (85.42)

According to the attitude survey, 91.67% of the trainees agreed that patient-centered care is crucial for treating chronic illnesses, indicating substantial support for this approach (Table 3). Patients should actively participate in their treatment choices, according to the majority (83.33%). Of the respondents, 75.75% expressed confidence in using professional guidelines for managing diabetes, while 67.71% said they were well-equipped to treat patients' hypertension. Furthermore, 88.54% of the respondents believed that treating chronic illnesses requires excellent patient communication.

**Table 3:** Attitudes Toward Patient-Centered Care in Chronic Disease Management

Attitude statement	Agree (number (%))
I believe patient-centered care is essential for managing chronic conditions.	350 (91.67)
I am confident in applying clinical guidelines to manage diabetes.	290 (75.75)
I believe patients should be actively involved in their treatment decisions.	320 (83.33)
I feel well-prepared to manage hypertension in patients.	260 (67.71)
I think that effective communication with patients is key in managing chronic diseases.	340 (88.54)

Regarding clinical procedures, 83.33% of the trainees consistently check blood pressure in patients with hypertension, and 78.13% regularly monitor blood glucose levels in patients with diabetes (Table 4). A sizable majority (80.83%) provide lifestyle modification counseling for the treatment of diabetes, while 79.38% do the same for hypertension. However, 72.92% of people with diabetes and 70.31% of those with hypertension reported following national standards for drug prescriptions, whereas 27.08% and 29.69%, respectively, did not regularly follow these criteria.

**Table 4:** Clinical Practices in Managing Diabetes and Hypertension

Practice question	Yes (number (%))	No (number (%))
Do you regularly monitor blood glucose levels in diabetic patients?	300 (78.13)	84 (21.87)
Do you regularly check blood pressure in hypertensive patients?	320 (83.33)	64 (16.67)
Do you provide counseling on lifestyle changes for managing diabetes?	310 (80.83)	74 (19.17)
Do you provide counseling on lifestyle changes for managing hypertension?	305 (79.38)	79 (20.63)
Do you adhere to national guidelines when prescribing medication for diabetes?	280 (72.92)	104 (27.08)
Do you adhere to national guidelines when prescribing medication for hypertension?	270 (70.31)	114 (29.69)

The relationship between the KAP levels of postgraduate medical trainees and a number of professional and demographic characteristics is shown in Table 5. Educational level (p-values of 0.015 for graduate (MBBS) and 0.029 for postgraduate (MD/MS)), clinical experience (p-value of 0.024 for 6-12 months), training program (p-value of 0.019 for general medicine), and age group (p-values of 0.015 for <25 years, 0.005 for 25-30 years, and 0.041 for >35 years) all showed significant associations. Gender, years of training, and clinical experience (13-24 months and >24 months) did not significantly correlate with KAP levels (p-values > 0.05).

**Table 5:** Association of Demographic and Professional Factors With KAP Levels Table 5 presents the chi-square (X²) values, p-values, and OR with 95% CI for the association between demographic and professional factors and KAP levels of postgraduate medical trainees. Significant associations (p-values < 0.05) are marked, with OR values indicating the strength of the relationship between each factor and higher KAP levels. Reference categories for OR calculations are specified with * in the table. KAP: knowledge, attitudes, and practices, MBBS: Bachelor of Medicine, Bachelor of Surgery, MD: Doctor of Medicine, MS: Master of Surgery, OR: odds ratios, CI: confidence interval

Variable		Chi-square (X²)	p-value	OR (95% CI)
Age group	<25 years	10.35	0.015	1.8 (1.2-2.6)
	25-30 years	12.42	0.005	2.1 (1.4-3.0)
	31-35 years	5.98	0.112	*
	>35 years	8.21	0.041	1.5 (1.1-2.2)
Gender	Male	1.21	0.271	-
	Female	1.67	0.196	-
Clinical experience	6-12 months	14.22	0.024	1.9 (1.3-2.8)
	13-24 months	7.89	0.117	1.2 (0.9-1.8)
	>24 months	3.56	0.168	*
Training program	General medicine	11.98	0.019	2.3 (1.5-3.4)
	Surgery	2.84	0.418	1.1 (0.8-1.7)
	Pediatrics	3.15	0.374	1.2 (0.9-1.9)
	Other	4.44	0.215	*
Educational level	Graduate (MBBS)	10.54	0.015	1.6 (1.2-2.4)
	Postgraduate (MD/MS)	9.03	0.029	*
Years of training	1-2 years	6.51	0.089	1.4 (1.0-2.0)
	3-4 years	7.76	0.1	1.3 (0.9-1.8)
	5+ years	2.97	0.225	*

## Discussion

The purpose of this research was to evaluate postgraduate medical trainees' knowledge of diabetes and hypertension treatment. The findings show that while trainees are typically well-versed in clinical standards and diagnostic criteria, there are still gaps in their practical application, especially when it comes to medication adherence and real-world management techniques.

According to the knowledge evaluation, 75.75% of the trainees comprehended the clinical recommendations for managing hypertension, and 83.33% were acquainted with those for managing diabetes. These results are in accordance with earlier research that showed that healthcare professionals knew a lot about clinical guidelines for managing chronic diseases [12]. As an illustration of the comparatively high understanding of clinical procedures among trainees, prior research by Ali et al. revealed that 80% of healthcare practitioners were aware of diabetes treatment recommendations [13]. Nonetheless, our analysis found that first-line drugs for hypertension (67.71%) and diabetes (70.31%) were considerably less well-known, indicating that treatment alternatives should be better understood. Similar findings were reported in a study by Wang et al., which highlighted knowledge gaps in medication use among healthcare providers managing hypertension and diabetes [14]. Additionally, Saleh demonstrated that while many practitioners were aware of diagnostic and lifestyle interventions, adherence to pharmacological recommendations was suboptimal [15].

The majority of the trainees (91.67%) agreed that patient-centered care is crucial for managing chronic conditions, which is consistent with other studies' findings that 89% of healthcare providers stressed the value of patient-centered care in managing chronic diseases [16]. Additionally, our survey showed a good movement toward collaborative care, with 83.33% of the trainees believing that patients should actively participate in their treatment choices. As seen by a prior research in which 80% of participants expressed similar opinions on patient involvement, this is corroborated by a worldwide trend in which healthcare providers are realizing the significance of patient engagement in treating chronic illnesses [17].

Improvement opportunities were also identified by clinical practices. Only 72.92% of the trainees reported following national recommendations when prescribing medicine for diabetes and 70.31% for hypertension, despite the fact that 78.13% of the trainees routinely examined blood glucose levels in diabetic patients and 83.33% evaluated blood pressure in hypertensive patients. These numbers are somewhat less than those of prior research that indicated that 85% of medical professionals followed prescription drug recommendations for diabetes [18]. High patient loads and resource constraints in clinical settings may have an impact on trainees' capacity to reliably adhere to clinical procedures, which might explain the disparity in guideline adherence.

Our study's correlation between KAP levels and professional and demographic characteristics reveals a number of noteworthy patterns. KAP levels were higher among younger trainees (less than 30 years old) and those with 6-12 months of clinical experience. This result is consistent with other studies that found early-stage trainees often had more positive attitudes and behaviors related to managing chronic diseases [19]. Nevertheless, no significant correlations between gender and KAP were discovered, which is in line with earlier research that indicated no gender disparities in healthcare practitioners' treatment of chronic illnesses [20].

Study strengths and limitations

The strength of this research is its thorough evaluation of postgraduate medical trainees' knowledge of managing diabetes and hypertension utilizing a large sample size of 384 participants from various clinical training programs. The dependability of the results is increased by the use of a validated questionnaire and strong statistical analysis. The research does, however, have a number of shortcomings. Because the study was only carried out at one institution, the findings may not be as applicable in other contexts. Furthermore, self-reported data could be skewed by answer biases, and the cross-sectional design precludes drawing conclusions about causality. Lastly, the research did not investigate how institutional resources and other external variables affect clinical practices.

## Conclusions

This research demonstrates that postgraduate medical trainees are highly committed to patient-centered care and have an in-depth understanding of clinical standards for controlling hypertension and diabetes. The quality of treatment may be impacted by the significant gaps in their actual implementation, especially with regard to medication adherence and consistent adherence to guidelines. The results highlight the need for focused enhancements in postgraduate education, with a focus on fusing academic understanding with practical clinical applications. Filling up these gaps might greatly improve patient outcomes in healthcare settings and the efficacy of managing chronic diseases.

## Appendices

The questionnaire used for data collection is shown in Table 6. 

**Table 6:** Questionnaire for Data Collection MBBS: Bachelor of Medicine, Bachelor of Surgery, MD: Doctor of Medicine, MS: Master of Surgery, BP: blood pressure

Section	Sub-sections	Question	Option 1	Option 2	Option 3	Option 4	Option 5
Demographics	Age group	What is your age group?	<25 years	25-30 years	31-35 years	>35 years	
	Gender	What is your gender?	Male	Female			
	Clinical experience	How many months of clinical experience?	6-12 months	13-24 months	>24 months		
	Training program	What is your clinical specialty?	General medicine	Surgery	Pediatrics	Other	
	Educational level	What is your highest educational level?	Graduate (MBBS)	Postgraduate (MD/MS)			
	Years of training	How many years of training?	1-2 years	3-4 years	5+ years		
Knowledge	Guidelines awareness	Are you aware of clinical guidelines for diabetes?	Yes	No			
	Diagnostic criteria	Are you familiar with hypertension diagnostic criteria?	Yes	No			
	Medication knowledge	Do you know the first-line medications for diabetes?	Yes	No			
	Lifestyle changes	Are you aware of lifestyle modifications for both conditions?	Yes	No			
Attitudes	Patient-centered care	Is patient-centered care essential?	Strongly agree	Agree	Neutral	Disagree	Strongly disagree
	Clinical confidence	Confident in using diabetes guidelines?	Yes	No			
	Patient involvement	Should patients participate in decisions?	Strongly agree	Agree	Neutral	Disagree	Strongly disagree
	Communication	Is effective communication key?	Strongly agree	Agree	Neutral	Disagree	Strongly disagree
Practices	Glucose monitoring	Do you regularly monitor blood glucose?	Yes	No			
	BP monitoring	Do you regularly check blood pressure?	Yes	No			
	Lifestyle counseling	Do you counsel diabetes lifestyle changes?	Yes	No			
	National guidelines	Do you follow guidelines for diabetes meds?	Yes	No			
	National guidelines	Do you follow guidelines for hypertension meds?	Yes	No			
